# Continuous persistent occiput transverse position during labor – associations of different midwifery interventions with cervical dilation rate and birth outcomes: A retrospective cohort study

**DOI:** 10.1097/MD.0000000000048574

**Published:** 2026-05-08

**Authors:** Mingjuan Jiang, Mingxia Zheng

**Affiliations:** a Obstetrics Department, Maternal and Child Health Care Hospital of Tongxiang City, Jiaxing City, Zhejiang Province, China.

**Keywords:** cervical dilation rate, labor progress, manual rotation, occiput transverse, retrospective cohort, rotational operative vaginal delivery

## Abstract

Persistent occiput transverse (OT) position during active labor can impede effective fetal descent and rotation, increasing the likelihood of labor dystocia and operative birth. Evidence comparing intrapartum management strategies often focuses on delivery mode, while cervical dilation rate – an important process metric guiding escalation – is less frequently evaluated. To compare different intrapartum management strategies for persistent OT in active labor with respect to cervical dilation rate and birth outcomes. We conducted a retrospective cohort study of term (≥37 + 0 weeks), singleton, cephalic pregnancies with persistent OT delivering between January 2023 and January 2025. Participants were classified by the primary strategy initiated after confirmation of persistent OT (index time): supportive care/positioning (n = 210), augmentation without rotation (n = 164), manual rotation attempt (n = 148), and planned rotational operative vaginal delivery (rOVD; n = 90). The primary outcome was cervical dilation rate (cm/h) from index to full dilation. Secondary outcomes included time to full dilation, mode of delivery, maternal morbidity (postpartum hemorrhage, obstetric anal sphincter injury [OASIS]), and neonatal outcomes. Multivariable regression models were applied, with supportive care as the reference; inverse probability of treatment weighting (IPTW) was prespecified as a sensitivity analysis. Mean dilation rate was highest in the manual rotation group (1.57 ± 0.39 cm/h) compared with supportive care (1.11 ± 0.31 cm/h; *P* < .001), with the shortest median time to full dilation (2.51 hours). In adjusted analyses, manual rotation was associated with a faster dilation rate (adjusted β + 0.423 cm/h, 95% CI: 0.358–0.488; *P* < .001) and lower odds of cesarean delivery (adjusted OR 0.51, 95% CI: 0.32–0.80; *P* = .004). OASIS was more frequent in the rOVD group (5.6%; *P* = .024). In this study, manual rotation was associated with the greatest improvement in cervical dilation rate and a favorable pattern of delivery outcomes. rOVD may facilitate delivery later in labor but may carry increased perineal morbidity. Prospective studies with standardized assessment and timing are warranted.

## 1. Introduction

Occiput transverse (OT) position is a common fetal head malposition encountered during labor. OT may be transient, and many fetuses rotate spontaneously to occiput anterior (OA) as descent occurs. Nevertheless, persistent OT in active labor – particularly in the second stage – can impede internal rotation and effective descent, predisposing to labor dystocia and escalation of intrapartum intervention.^[1]^ Risk factors for persistent OT include nulliparity, maternal obesity, increased birth weight, and epidural analgesia.^[2]^ The clinical significance of this condition lies in the biomechanical mismatch it creates: the fetal head presents with its longer diameters (e.g., occipitofrontal or biparietal) aligned unfavorably relative to the maternal pelvis, impeding rotation and descent, and predisposing to protracted labor, arrest, and operative delivery.^[3]^

Persistent malposition is important not only as an anatomic description but also as a determinant of labor efficiency and maternal outcomes. Management decisions occur within an evolving understanding of “normal” labor progress. Contemporary labor curve analyses from the Consortium on Safe Labor demonstrated substantial variation in cervical dilation patterns and suggested that acceleration often occurs later than traditionally taught; in particular, slower progress before 6 cm may still be consistent with normal outcomes.^[2]^ In line with these data, the American College of Obstetricians and Gynecologists (ACOG) updated guidance on first- and second-stage labor management, including refined definitions for protraction and arrest and recommendations intended to support judicious timing of interventions that may influence cesarean delivery rates.^[3]^ Within this framework, cervical dilation rate becomes a clinically meaningful process indicator: it is measurable, closely tied to escalation decisions, and may help distinguish interventions that truly improve labor physiology from those that primarily alter the delivery endpoint.

In clinical practice, management of persistent OT spans supportive, augmentation, and corrective approaches. Supportive care typically includes maternal positioning, mobility, continuous labor support, and comfort measures intended to facilitate spontaneous rotation and optimize pelvic mechanics. Randomized trials of postural interventions in malposition have reported mixed results. In a randomized controlled trial, hands-and-knees positioning improved maternal comfort but did not consistently increase OA at birth.^[4]^ Similarly, another randomized trial evaluating maternal positioning during the first stage did not demonstrate a clear increase in correction of malposition, despite potential benefits for maternal experience.^[5]^ Collectively, these findings suggest that although supportive strategies are generally low-risk and may improve comfort, they may be insufficient for a subset of labors with persistent malposition.

Augmentation strategies – amniotomy and/or oxytocin – are commonly used to optimize uterine activity and may facilitate descent and rotation, but they are often applied in response to slower progress, making causal effects difficult to separate from the underlying indication. Manual rotation represents a more direct corrective strategy aimed at converting OT (or occiput posterior) to OA. Observational evidence indicates that successful manual rotation is associated with lower cesarean delivery rates compared with failed attempts and has identified predictors of success linked to parity and intrapartum conditions.^[6]^ Randomized evidence has also emerged. The PROPOP randomized clinical trial evaluated a prophylactic attempt of manual rotation for OP/OT in early second stage and reported reductions in operative delivery and shorter second-stage duration in the intervention group.^[7]^ Furthermore, a systematic review and meta-analysis of randomized controlled trials found that manual rotation increased spontaneous vaginal delivery overall, with no clear signal of worse neonatal outcomes, although effect estimates varied by trial design and clinical context.^[8]^

When malposition persists late in labor or expedited birth is indicated, rotational operative vaginal delivery (rOVD) may be considered to achieve vaginal birth while avoiding second-stage cesarean, provided prerequisites are met and appropriately skilled operators are available.^[9]^ Royal College of Obstetricians and Gynaecologists (RCOG) guidance emphasizes that clinicians should develop competency in at least 1 specialist rotational technique and that senior support and governance are essential for safe rotational or midpelvic assisted births.^[10]^ At the same time, rotational techniques can be associated with maternal soft tissue injury, underscoring the importance of careful candidate selection, technique, and outcome reporting.

Despite increasing evidence on delivery mode and selected complications, fewer studies have evaluated how different intrapartum management strategies – including maternal positioning, oxytocin infusion, manual rotation, and rotational operative vaginal delivery (rOVD) – influence cervical dilation rate among women with persistent OT. This gap is notable because dilation rate serves as a key process indicator that guides clinical escalation and helps distinguish interventions that truly improve labor physiology from those that primarily alter the delivery endpoint. Moreover, comparative effectiveness analyses in routine care are vulnerable to confounding by indication: slower progress, fetal station, analgesia, uterine activity, and fetal status influence both treatment selection and outcomes. Without appropriate adjustment, observational comparisons may overestimate benefits or harms. Accordingly, we aimed to compare these intrapartum management strategies for persistent OT position in active labor, focusing on their impact on first-stage progression (cervical dilation rate) as well as delivery outcomes (spontaneous vaginal birth, operative vaginal birth, and cesarean delivery).

## 2. Materials and methods

### 2.1. Study design

This study was approved by the Ethics Committee of Maternal and Child Health Care Hospital of Tongxiang City (approval No. MC[2023]011, approved on January 10, 2023). This retrospective cohort study enrolled women who delivered at our institution between (January 2023) and (January 2025). Eligible participants were term (≥37 + 0 weeks) singleton pregnancies with cephalic presentation who entered active labor and were diagnosed with persistent occiput transverse (OT) position during labor. Participants were classified into 4 exposure groups according to the primary intrapartum management strategy initiated after confirmation of persistent OT: supportive care/positioning, labor augmentation (amniotomy and/or oxytocin) without rotation attempt, manual rotation attempt, and planned rotational operative vaginal delivery (rOVD). Group assignment was determined by documented intrapartum records (partogram, midwifery notes, operative notes) and followed an intention-to-treat-by-strategy principle.

Women were required to have complete intrapartum time stamps and cervical examination records sufficient to calculate cervical dilation rate from the index time to full dilation. Exclusion criteria included multiple gestation, major fetal anomalies, planned pre-labor cesarean delivery, and incomplete or missing key variables preventing exposure classification or primary outcome calculation. The study was conducted in accordance with the Declaration of Helsinki. Because of the retrospective design and use of de-identified clinical data, written informed consent was waived (or obtained, depending on your IRB requirement).

### 2.2. Diagnostic criteria for persistent OT

In this study, persistent OT was defined using an operational intrapartum criterion: OT position documented on 2 consecutive assessments separated by at least 60 minutes during active labor (cervical dilation ≥ 5 cm), or OT documented in early second stage prior to any rotation attempt, based on routine clinical assessment. Fetal head position was primarily assessed by vaginal examination and, when performed as part of unit practice, confirmed by intrapartum ultrasound.

To minimize misclassification, cases were not assigned when records described only “suspected OT” without a clear documented position, or when malposition was noted once but subsequently recorded as OA within the prespecified interval without meeting the persistence definition. If inconsistent position documentation occurred, predefined adjudication rules were applied (e.g., preference for contemporaneous detailed examinations; exclusion when persistence could not be determined). The index time (“time zero”) was defined as the first time point meeting the persistence criteria and was used to anchor exposure ascertainment and outcome calculations.

### 2.3. Data collection

Clinical data were retrospectively extracted from electronic medical records and standardized intrapartum documentation for deliveries occurring between (January 2023) and (January 2025). The primary outcome was cervical dilation rate from the index time to full dilation, calculated using prespecified rules. Data collection covered 4 domains.

Maternal and baseline characteristics included age, body mass index (BMI; prepregnancy or admission), parity (nulliparous vs multiparous), gestational age at delivery, and labor onset type (spontaneous vs induction).

Intrapartum characteristics at index time included cervical dilation (cm), fetal station, membrane status, fetal heart rate abnormality around index time (when recorded), and relevant clinical concerns such as suspected cephalopelvic disproportion (when documented).

Intrapartum interventions included epidural analgesia, oxytocin exposure (prior to index time and/or initiated/escalated after index time), amniotomy, manual rotation attempt (yes/no; time and documentation), and planned rOVD (device/technique where available).

Outcomes included time from index to full dilation, second-stage duration, mode of delivery (spontaneous vaginal, operative vaginal, cesarean), postpartum hemorrhage (PPH; per institutional definition, e.g., ≥1000 mL), obstetric anal sphincter injury (OASIS; third–fourth degree), intrapartum fever/clinical chorioamnionitis, and neonatal outcomes (5-minute Apgar < 7, NICU admission, and umbilical artery pH < 7.10 when available).

Two investigators independently verified extracted variables against source records; discrepancies were resolved by consensus with a senior reviewer. Continuous variables were recorded in original units, and categorical variables were coded using predefined categories to ensure consistency.

### 2.4. Statistical analysis

All analyses were performed using IBM SPSS Statistics (version 27.0). Continuous variables are presented as mean ± SD or median (IQR), as appropriate, and categorical variables as n (%). Group comparisons used 1-way ANOVA (Welch ANOVA if variances were unequal) or Kruskal–Wallis tests for continuous variables, and chi-square or Fisher exact tests for categorical variables.

For the primary outcome, multivariable linear regression estimated associations between management strategy and cervical dilation rate (cm/h), using supportive care/positioning as the reference; results are reported as adjusted β with 95% CIs. Binary secondary outcomes were analyzed using multivariable logistic regression and reported as adjusted odds ratios (aORs) with 95% CIs. Time-to-event outcomes were evaluated using Cox proportional hazards models when applicable, with cesarean delivery before full dilation handled as prespecified (e.g., censored at cesarean time).

To address confounding by indication, inverse probability of treatment weighting (IPTW) using multinomial propensity scores was conducted as a prespecified sensitivity analysis. Covariate balance was assessed using standardized mean differences (SMD), with SMD < 0.10 indicating adequate balance; weighted models used robust standard errors. Missing data were summarized and, when nontrivial, handled using multiple imputation by chained equations under a missing-at-random assumption, with results compared to complete-case analyses. All tests were 2-sided with *P* < .05 considered statistically significant.

## 3. Results

### 3.1. Study population and missing data

A total of 612 women with persistent occiput transverse (OT) position in active labor were included and classified by the primary management strategy initiated after confirmation of persistent OT: supportive care/positioning (n = 210), augmentation without rotation attempt (n = 164), manual rotation attempt (n = 148), and planned rotational operative vaginal delivery (rOVD; n = 90).

Missingness was low for most covariates: BMI 3.8% (23/612) and epidural 1.6% (10/612). Umbilical artery pH was unavailable for 18.1% (111/612).

### 3.2. Baseline characteristics

Baseline characteristics at the index time are summarized in Table 1. Groups were broadly similar in age, BMI, gestational age, parity, induction, epidural use, and intrapartum risk markers (meconium, suspected CPD, and fetal heart rate abnormality). As expected clinically, the rOVD group entered the strategy window at a later cervical dilation (mean 7.0 ± 0.7 cm) compared with the supportive, augmentation, and manual rotation groups (means ~6.2–6.3 cm, *P* < .001). Fetal station distributions at index time were not significantly different across groups (*P* = .572; Table 1).

**Table 1:** Baseline characteristics at index time.

Characteristic	Supportive (n = 210)	Augmentation (n = 164)	Manual rotation (n = 148)	rOVD (n = 90)	*P* value
Maternal age, yr	31.1 (4.4)	31.0 (4.9)	30.6 (4.4)	31.3 (3.8)	.597
BMI, kg/m^2^	26.5 (3.9)	26.5 (4.2)	26.5 (3.4)	26.4 (3.7)	.992
Gestational age, wk	39.0 (1.0)	39.2 (1.0)	38.9 (1.0)	39.0 (1.0)	.262
Cervical dilation at index, cm	6.3 (0.8)	6.2 (0.8)	6.2 (0.8)	7.0 (0.7)	<.001
Age ≥ 35 yr	43/210 (20.5%)	39/164 (23.8%)	26/148 (17.6%)	15/90 (16.7%)	.448
BMI ≥ 30 kg/m^2^	41/203 (20.2%)	34/159 (21.4%)	23/145 (15.9%)	14/87 (16.1%)	.535
Gestational age ≥ 41 wk	8/210 (3.8%)	6/164 (3.7%)	5/148 (3.4%)	3/90 (3.3%)	.995
Nulliparous	153/210 (72.9%)	122/164 (74.4%)	112/148 (75.7%)	61/90 (67.8%)	.584
Induction of labor	59/210 (28.1%)	39/164 (23.8%)	43/148 (29.1%)	24/90 (26.7%)	.726
Epidural analgesia	130/209 (62.2%)	94/161 (58.4%)	90/147 (61.2%)	55/90 (61.1%)	.901
Ruptured membranes at index	127/210 (60.5%)	85/164 (51.8%)	86/148 (58.1%)	61/90 (67.8%)	.088
Oxytocin exposure prior to index	64/210 (30.5%)	36/164 (22.0%)	34/148 (23.0%)	17/90 (18.9%)	.098
Meconium-stained fluid	18/210 (8.6%)	22/164 (13.4%)	26/148 (17.6%)	12/90 (13.3%)	.091
Suspected CPD	17/210 (8.1%)	15/164 (9.1%)	18/148 (12.2%)	13/90 (14.4%)	.312
FHR abnormality at index	28/210 (13.3%)	24/164 (14.6%)	23/148 (15.5%)	14/90 (15.6%)	.932
**Fetal station distribution at index time (*P* = .572**)					
Station at index	Supportive	Augmentation	Manual rotation	rOVD	
−2	21 (10.0%)	19 (11.6%)	18 (12.2%)	9 (10.0%)	
−1	62 (29.5%)	57 (34.8%)	44 (29.7%)	25 (27.8%)	
0	72 (34.3%)	48 (29.3%)	45 (30.4%)	22 (24.4%)	
+1	39 (18.6%)	22 (13.4%)	27 (18.2%)	21 (23.3%)	
+2	16 (7.6%)	18 (11.0%)	14 (9.5%)	13 (14.4%)	

BMI = body mass index, CPD = cephalopelvic disproportion, FHR = fetal heart rate, rOVD = rotational operative vaginal delivery.

### 3.3. Labor progress outcomes

Labor progress outcomes are shown in Table 2. The manual rotation group had the highest mean cervical dilation rate (1.57 ± 0.39 cm/h) and the shortest median time from index to full dilation (2.51 hours). Differences across groups were significant for dilation rate (*P* < .001), time to full dilation (*P* < .001), and second-stage duration (*P* < .001).

**Table 2:** Labor progress outcomes.

Group	Cervical dilation rate, cm/h Mean (SD)	Time to full dilation, h Median (IQR)	Second stage, min Median (IQR)
Supportive	1.11 (0.31)	3.40 (2.96–3.89)	86 (71–103)
Augmentation	1.29 (0.37)	3.06 (2.71–3.38)	86 (69–105)
Manual rotation	1.57 (0.39)	2.51 (2.20–2.97)	76 (56–88)
rOVD	1.18 (0.34)	2.64 (2.31–2.97)	68 (51–82)

IQR = interquartile range, rOVD = rotational operative vaginal delivery, SD = standard deviation.

### 3.4. Mode of delivery and maternal outcomes

Mode of delivery differed significantly by strategy (overall *P* < .001; Table 3). post hoc pairwise comparisons revealed that the manual rotation group had a significantly lower cesarean delivery rate compared with the supportive care group (39.9% vs 54.8%, *P* = .003) and the augmentation group (39.9% vs 52.4%, *P* = .021), while no significant difference was observed between the manual rotation and rOVD groups (39.9% vs 50.0%, *P* = .146). Operative vaginal delivery was most frequent in the rOVD group (40.0%) and the manual rotation group (26.4%).

**Table 3:** Delivery mode and maternal outcomes.

Outcome	Supportive	Augmentation	Manual rotation	rOVD	*P* value
Spontaneous vaginal	80 (38.1%)	59 (36.0%)	50 (33.8%)	9 (10.0%)	
Operative vaginal	15 (7.1%)	19 (11.6%)	39 (26.4%)	36 (40.0%)	
Cesarean	115 (54.8%)	86 (52.4%)	59 (39.9%)	45 (50.0%)	
PPH ≥ 1000 mL	17/210 (8.1%)	10/164 (6.1%)	11/148 (7.4%)	7/90 (7.8%)	.902
OASIS (third–fourth degree)	1/210 (0.5%)	3/164 (1.8%)	2/148 (1.4%)	5/90 (5.6%)	.024
Intrapartum fever/chorio	21/210 (10.0%)	17/164 (10.4%)	12/148 (8.1%)	5/90 (5.6%)	.556

Overall mode-of-delivery distribution *P* < .001.

OASIS = obstetric anal sphincter injury, PPH = postpartum hemorrhage, rOVD = rotational operative vaginal delivery.

Maternal complications were uncommon overall. OASIS differed significantly across groups (*P* = .024). post hoc comparisons showed that the rOVD group had a significantly higher OASIS rate compared with the supportive care group (5.6% vs 0.5%, *P* = .018) and the augmentation group (5.6% vs 1.8%, *P* = .046), while the difference between rOVD and manual rotation groups did not reach statistical significance (5.6% vs 1.4%, *P* = .081). PPH and intrapartum fever/chorioamnionitis did not differ significantly across groups.

### 3.5. Neonatal outcomes

Neonatal outcomes are summarized in Table 4. Rates of 5-minute Apgar < 7 did not differ significantly across groups (*P* = .741). NICU admission differed significantly by strategy (*P* = .040). post hoc pairwise comparisons revealed that the supportive care group had a significantly higher NICU admission rate compared with the augmentation group (9.5% vs 4.3%, *P* = .049) and the manual rotation group (9.5% vs 4.7%, *P* = .045), while the difference between supportive care and rOVD groups did not reach statistical significance (9.5% vs 2.2%, *P* = .056). No significant differences were observed among the augmentation, manual rotation, and rOVD groups. Umbilical artery pH was available in 81.9% overall; no recorded samples met pH < 7.10 in this cohort.

**Table 4:** Neonatal outcomes.

Outcome	Supportive	Augmentation	Manual rotation	rOVD	*P* value
5-min Apgar < 7	7/210 (3.3%)	4/164 (2.4%)	7/148 (4.7%)	3/90 (3.3%)	.741
NICU admission	20/210 (9.5%)	7/164 (4.3%)	7/148 (4.7%)	2/90 (2.2%)	.040
Umbilical artery pH available	171/210 (81.4%)	133/164 (81.1%)	119/148 (80.4%)	78/90 (86.7%)	
Umbilical artery pH, mean (SD)	7.24 (0.04)	7.24 (0.04)	7.24 (0.04)	7.25 (0.04)	
Umbilical artery pH < 7.10	0/171 (0.0%)	0/133 (0.0%)	0/119 (0.0%)	0/78 (0.0%)	

NICU = neonatal intensive care unit, rOVD = rotational operative vaginal delivery, SD = standard deviation.

### 3.6. Multivariable analyses

Complete-case multivariable models included 589 women. After adjustment for age, BMI, parity, gestational age, induction, epidural, cervical dilation and station at index time, membrane status, oxytocin prior to index time, meconium, suspected CPD, and fetal heart rate abnormality, all active strategies were associated with a higher cervical dilation rate compared with supportive care (Table 5). The largest adjusted effect was observed for manual rotation (adjusted β + 0.423 cm/h, 95% CI: 0.358–0.488, *P* < .001). Manual rotation was also associated with lower odds of cesarean delivery (adjusted OR 0.51, 95% CI: 0.32–0.80, *P* = .004). For OASIS, effect estimates were imprecise due to few events, with the highest point estimate in rOVD.

**Table 5:** Adjusted associations vs supportive care (n = 589).

Strategy vs supportive	Adjusted β for dilation rate (cm/h) (95% CI)	*P*	Adjusted OR for cesarean (95% CI)	*P*	Adjusted OR for OASIS (95% CI)	*P*
Augmentation	0.134 (0.078–0.191)	<.001	0.92 (0.59–1.44)	.722	4.34 (0.42–45.19)	.220
Manual rotation	0.423 (0.358–0.488)	<.001	0.51 (0.32–0.80)	.004	2.42 (0.27–21.83)	.430
rOVD	0.244 (0.177–0.312)	<.001	0.80 (0.47–1.37)	.411	11.39 (0.62–209.52)	.102

BMI = body mass index, CI = confidence interval, OR = odds ratio, rOVD = rotational operative vaginal delivery.

## 4. Discussion

In this retrospective cohort of women with persistent occiput transverse (OT) position during active labor, we compared 4 pragmatic management pathways – supportive care/positioning, augmentation without rotation, manual rotation attempt, and planned rotational operative vaginal delivery (rOVD) – using cervical dilation rate as a clinically decision-relevant process outcome alongside delivery and neonatal endpoints. The most consistent pattern was that manual rotation was associated with the fastest cervical dilation rate and shorter time to complete dilation, whereas augmentation showed a smaller improvement relative to supportive care. The directionality is biologically plausible and aligns with contemporary understanding that persistent malposition can impair effective descent and rotation, thereby reducing the efficiency of uterine forces and slowing progress. Current professional guidance emphasizes careful assessment of labor progress, avoiding premature diagnosis of arrest, and considering appropriately selected interventions (including rotation techniques and operative vaginal birth) when indicated.^[11,12]^

A key contribution of this manuscript framework is the explicit use of dilation rate rather than relying only on final delivery mode. Labor curve research from large contemporary datasets demonstrates substantial heterogeneity in “normal” labor patterns and cautions against rigid expectations, especially when using historical thresholds that may lead to earlier diagnosis of arrest and unnecessary cesarean delivery.^[13]^ In the setting of persistent OT, dilation rate may serve as an “early” indicator capturing whether an intervention meaningfully improves the mechanics of labor (e.g., improved head alignment and descent after successful rotation) before downstream decisions about operative birth are made. In practice, clinicians often escalate from supportive measures to augmentation and then to more direct correction (manual rotation) or operative delivery depending on maternal–fetal status and progress; a process metric like dilation rate mirrors that real-time decision logic more directly than mode of birth alone.

The advantage observed with manual rotation is consistent with observational evidence suggesting that rotation success is associated with higher vaginal birth likelihood and lower cesarean rates, and that intrapartum conditions (such as dilation, station, and indication) influence the probability of success.^[14,15]^ Trial-level evidence is more heterogeneous, but recent randomized and synthesis work supports potential benefit in selected populations. The PROPOP randomized clinical trial evaluated prophylactic manual rotation for occiput posterior/transverse malpositions in early second stage and provides important methodological precedent for defining timing, standardizing technique, and monitoring safety.^[16]^ A systematic review and meta-analysis of randomized controlled trials also reports increased spontaneous vaginal birth with manual rotation, although certainty may vary by setting, operator experience, and implementation fidelity.^[17]^ The TURN-OUT study protocol further illustrates how investigators conceptualize OT as a modifiable intrapartum condition and highlights practical design issues (case ascertainment, timing, and outcome selection) that are also relevant to observational analyses.^[18]^ Together, these published data support a clinically coherent hypothesis: when persistent OT is a major contributor to inefficient labor mechanics, correcting malposition (rather than only strengthening contractions) may have a larger effect on progress.

Augmentation was associated with modestly improved dilation rate compared with supportive care in the study, which matches a common clinical pathway – optimize uterine activity before attempting more operator-dependent maneuvers. However, if malposition is the dominant mechanism, augmentation alone may have limited ability to overcome unfavorable head–pelvis relationships. Supportive strategies, including maternal positioning, are widely used because they are low-risk and acceptable to many women, and they may improve comfort even when they do not materially change fetal rotation. Randomized trials of hands-and-knees or similar positions for malposition have generally not demonstrated large improvements in rotation to occiput anterior or major reductions in operative birth, despite potential benefits for maternal comfort.^[19,20]^ Although OT and occiput posterior (OP) are not identical, both involve malrotation dynamics that can become entrenched as labor advances; therefore, it is plausible that positioning alone may be insufficient once persistent malposition is established during active labor.

For rOVD, the pattern – later timing (greater dilation at index), high operative vaginal birth proportion, and a signal toward more obstetric anal sphincter injury (OASIS) – reflects real-world trade-offs in rotational instrumental birth. Operative vaginal birth guidance stresses that rOVD requires operator expertise, appropriate setting and support, and careful case selection to balance timely delivery against maternal pelvic floor trauma.^[20]^ Persistent malposition (especially OP, often used as an analog for difficult rotation) has been associated with increased operative delivery and perineal trauma in cohort studies, reinforcing that malposition can be a marker of more challenging mechanics and higher intervention intensity.^[21,22]^ These considerations support a stepwise approach in which manual rotation – when safe and feasible – may be considered before proceeding to rotational instrumentation, while acknowledging that rOVD remains an important option when maternal–fetal urgency, station, or labor stage limits alternatives.

Methodologically, comparisons across strategies are vulnerable to confounding by indication: slow progress triggers interventions, and advanced labor stage may prompt rOVD. Although multivariable adjustment and IPTW can reduce measured confounding, unmeasured factors (operator skill, caput/molding, nuanced fetal head engagement assessment, unit culture) may still bias estimates. A related issue is misclassification of fetal head position when assessed by digital examination alone. Intrapartum ultrasound improves accuracy of fetal head position assessment, particularly before instrumental delivery, and may therefore improve both clinical decision-making and research validity when incorporated into standardized protocols.^[14]^ Finally, fetal position is dynamic: classic work documenting changes in fetal head position during labor and exploring associations with epidural analgesia highlights the importance of repeated assessments and the potential for time-varying confounding in observational studies.^[23]^ Future research – ideally prospective, with ultrasound-confirmed position, standardized “index time” definitions, and documentation of operator experience – could strengthen causal inference, while also incorporating process outcomes (dilation rate, time-to-full dilation) alongside traditional endpoints.^[24]^

Neonatal outcomes in the study were broadly similar across groups, which is reassuring and aligns with the concept that many cases of malposition can be managed safely when fetal status is monitored and timely escalation occurs. Nonetheless, persistent malposition at delivery (particularly OP) has been associated with higher risk of adverse neonatal outcomes in large retrospective cohorts, underscoring the importance of surveillance, timely management, and appropriate counseling.^[25]^ Even when interventions aim to reduce cesarean delivery, safety endpoints – including neonatal acid–base status, Apgar scores, and NICU admission – remain essential to interpret net benefit.

### 4.1. Limitations

Several limitations should be acknowledged. The retrospective, single-center design limits generalizability. Cervical dilation and fetal head position were assessed by digital examination, introducing subjective measurement variability; intrapartum ultrasound was not uniformly used. Despite adjustment for measured confounders, unmeasured factors – including operator experience in performing rotational procedures and prenatal fetal wellbeing status (e.g., Doppler findings, amniotic fluid volume, estimated fetal weight) – may still influence outcomes. Additionally, causal inferences cannot be drawn from this observational study. Future prospective, multicenter studies with standardized protocols and ultrasound confirmation are warranted.

## 5. Conclusion

In this study of persistent OT during active labor, manual rotation was associated with the greatest improvement in cervical dilation rate and a favorable pattern of delivery outcomes compared with supportive care, while augmentation offered more modest acceleration. Planned rOVD appeared to facilitate delivery in later labor but with a potential trade-off in perineal morbidity. These findings support a pragmatic staged strategy consistent with contemporary guidance: confirm malposition (preferably with ultrasound when available), optimize supportive care and uterine activity, and consider timely manual rotation in appropriate candidates before progression to rotational operative delivery. Prospective studies that standardize timing/technique of rotation and incorporate labor process metrics (including dilation rate) are warranted.

## Author contributions

**Conceptualization:** Mingjuan Jiang, Mingxia Zheng.

**Data curation:** Mingjuan Jiang, Mingxia Zheng.

**Formal analysis:** Mingjuan Jiang, Mingxia Zheng.

**Funding acquisition:** Mingjuan Jiang, Mingxia Zheng.

**Investigation:** Mingjuan Jiang, Mingxia Zheng.

**Writing – original draft:** Mingxia Zheng.

**Writing – review & editing:** Mingxia Zheng.
